# Aptamer-based Field-Effect Biosensor for Tenofovir Detection

**DOI:** 10.1038/srep44409

**Published:** 2017-03-15

**Authors:** N. Aliakbarinodehi, P. Jolly, N. Bhalla, A. Miodek, G. De Micheli, P. Estrela, S. Carrara

**Affiliations:** 1 School of Engineering, École Polytechnique Fédérale de Lausanne (EPFL), STI-IEL-LSI2, Building INF, 3rd floor, 1015 Lausanne, Switzerland; 2 Department of Electronic and Electrical Engineering, University of Bath, Claverton Down, BA2 7AY Bath, United Kingdom

## Abstract

During medical treatment it is critical to maintain the circulatory concentration of drugs within their therapeutic range. A novel biosensor is presented in this work to address the lack of a reliable point-of-care drug monitoring system in the market. The biosensor incorporates high selectivity and sensitivity by integrating aptamers as the recognition element and field-effect transistors as the signal transducer. The drug tenofovir was used as a model small molecule. The biointerface of the sensor is a binary self-assembled monolayer of specific thiolated aptamer and 6-mercapto-1-hexanol (MCH), whose ratio was optimized by electrochemical impedance spectroscopy measurements to enhance the sensitivity towards the specific target. Surface plasmon resonance, performed under different buffer conditions, shows optimum specific and little non-specific binding in phosphate buffered saline. The dose-response behavior of the field-effect biosensor presents a linear range between 1 nM and 100 nM of tenofovir and a limit of detection of 1.2 nM. Two non-specific drugs and one non-specific aptamer, tested as stringent control candidates, caused negligible responses. The applications were successfully extended to the detection of the drug in human serum. As demonstrated by impedance measurements, the aptamer-based sensors can be used for real-time drug monitoring.

Label-free therapeutic drug monitoring (TDM) by means of electrochemical^1^ and field-effect biosensors^2^ has gathered significant interest in the last decade since it can lead to personalized point-of-care (POC) therapy applications^3^,^4^,^5^,^6^. The main advantages of electrochemical and field-effect biosensors over traditional optical techniques for TDM are their availability, cost effectiveness, simplicity and portability. Drug biosensors are key elements in bringing treatments to the patient’s bedside. They have completely changed and improved the traditional dosing regimen to a mechanism that administers the medication based on the patients’ characteristics (genetic profile, age, race, gender, environmental agents, etc.) and drug influences, including pharmacodynamics, pharmacokinetics, drug-drug interactions, adverse effects, etc^7^,^8^,^9^,^10^,^11^. In other words, patients take advantage of point-of-care biosensors to control the circulating drug concentration in their body over time themselves, and administer the optimum dosage of drugs in need.

For the continuous monitoring of therapeutic compounds, enzymatic biosensors that utilize cytochrome P450 (CYP450) as specific bio-probes and amperometric detection have been developed as drug detection systems thanks to their simplicity and their compliance with point of care TDM requirements^12^. CYP450-based electrochemical sensors present an optimal sensitivity and detection limit that addresses therapeutic ranges for many drugs, especially once coupled with multi-walled carbon nanotubes^13^,^14^,^15^. They enable lower selectivity with respect to affinity-based biosensors considering that each CYP450 enzyme can have a wide range of substrates or inhibitors. However, the lifetime of such biosensors is still fairly short, thus limiting their application range in continuous monitoring^12^. The need for more robust electrochemical biosensors for continuous drugs monitoring that picks out one specific target is still not yet fully addressed. To overcome the above-mentioned issues, an n-channel metal-oxide semiconductor field-effect transistor device (n-MOSFET) with an extended gate functionalized with a drug-specific DNA aptamer is used in this work. The gate of the transistor is physically connected to an external gold electrode, which is used for functionalization with an aptamer specific to a drug and exposed to the measurement sample. When the potential applied across the gate and the source (V_gs_) is larger than the threshold voltage (V_th_) of the device (V_gs_ > V_th_), a conducting channel is formed between the source and the drain. Additionally, if a voltage is applied between the drain and source (V_ds_ > 0), then a current (I_d_) begins to flow through the induced channel under the gate dielectric. This condition of the device is known as turn-on state. Any changes across V_gs_ can modulate the conductivity of the channel and alter I_d_. If molecular interactions take place at the gate of the transistor, such as when negatively charged small molecules (for instance drugs) are captured by recognition probes (aptamers in this work), the minimum V_gs_ required to bring the n-MOSFET to the turn-on state is increased (gating effect). This is used to quantify the binding event by monitoring the shift of the transfer characteristic (I_d_ *vs.* V_gs_ curve) of the transistor after the target molecule binding^16^,^17^.

Aptamers are short oligonucleotide sequences that can strongly bind to their targets with high affinity and specificity thanks to specific conformational changes. Aptamers have several advantages with respect to antibodies. For instance, selection of aptamers is performed through an *in vitro* process. Once the aptamers are selected, they can be further synthesized in a controlled fashion with high purity and reproducibility. Additionally, aptamers are chemically more stable and they retain most of their functionality even after multiple regeneration steps^18^,^19^. Such a regeneration step is advantageous for continuous monitoring studies. For example, Ferguson *et al*.^4^ have proposed a novel selective and sensitive real-time small molecule monitoring method that uses aptamers as recognition elements, and prevents the interaction of blood molecules with the sensing surface by a continuous flow diffusion filter in order to suppress sensor fouling. As oligonucleotides can be easily modified with different reactive chemical groups, their immobilization on surfaces can be easily controlled. Thanks to such flexibility, aptamers are very popular for the design and optimization of novel biosensors^20^. Such probes, e.g. DNA aptamers, are gaining significant attention for the detection of several small molecules^21^.

As a case study for the development of a biosensor for drug monitoring, the targeted drug tenofovir (TFV) was used in the current work. TFV is an antiretroviral drug that was approved by the FDA in 2001 for medical prevention and treatment of patients with human immunodeficiency virus (HIV) infections and treatments for hepatitis-B. Electrochemical biosensors for small molecule sensing have been investigated for the last forty years^22^, yet a reliable system has not been characterized completely, to the best of our knowledge. Therefore, a highly selective and sensitive aptamer based biosensor for continuous monitoring of drugs in buffer and plasma is proposed in our work (AptaFET). This biosensor is based on the combination of aptamers for target recognition and field-effect transistors for the transduction of the drug-probe interaction to an electric signal (schematic and more detail in Supplementary Material, Figure S3).

## Results

The sensor response to specific and non-specific drug−aptamer interaction was measured by surface plasmon resonance (SPR) at different buffer conditions. Electrochemical impedance spectroscopy (EIS) was used to optimize the biosensing interface by finding the best ratio between thiolated aptamer and MCH concentrations used on the immobilization process. To obtain the dose-response behavior of the system, the AptaFET biosensor was measured upon interaction with different concentrations of TFV in PBS buffer. Control experiments were carried out using Abi and Enza (non-specific drugs) and a PSA-aptamer (non-specific aptamer). AptaFET application for drug monitoring in human plasma was investigated by detecting 500 nM of TFV in human plasma. Real time impedance was used to investigate the performance of the sensing interface under flow conditions enabling the application of the biosensor in real time drug monitoring.

### Binding reaction investigation

**Figure 1:**
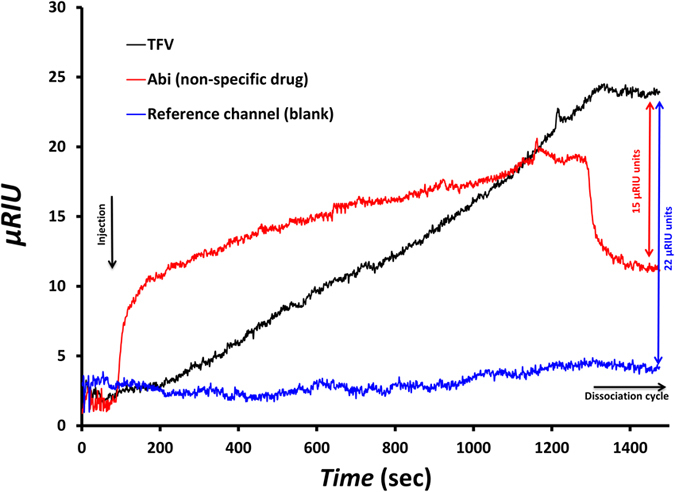
Specific interaction of TFV and TFV-aptamer (black line), and non-specific interaction of Abi with TFV-aptamer (red line). Blank response is illustrated in blue. A 15 μRIU difference was recorded between specific and non-specific interactions.

Surface Plasmon Resonance (SPR) experiments were carried out as a primary study utilizing two parallel channels simultaneously on each biofunctionalized chip in order to verifiy the functionalization of the sensing surface. Binding of targets to the sensing surface was studied using SPR experiment at different buffer conditions, with one channel used for drug spiked sample and the other one for blank buffer. The sensing surface was modified with MCH and TFV specific aptamer (TFV-aptamer) as described in the bioelectrode development section. TFV and abiraterone (Abi) solutions with concentrations of 500 nM were injected and the variations of reflectivity angle were monitored during the incubation cycle. This was followed by washing steps to remove all non bonded residues. Abi (a prostate cancer drug) was used as a negative control to study the specificity of the detection. Experiments were carried out in phosphate buffer (PB; pH 7.0), phosphate buffer saline (PBS; 10 mM; pH 7.4) and PBS containing 1 mM MgCl_2_ (PBS-MgCl_2_). A washing step was carried out using the same buffer but without the drug. The recorded signals exhibited much lower target binding effect for experiments performed in PB and PBS with respect to PBS-MgCl_2_ (Fig. 1), in which binding reactions were clearly observed for both TFV (as target molecule) and Abi. SPR response showed positive binding interaction for both experimented drugs. This was observed as an increment of refractive index unit (RIU) in the SPR signal. Nevertheless, Abi molecules were removed from the surface during the dissociation cycle, while TFV molecules remained bound to the aptamers. After 20 minutes of incubation specific interaction of aptamer/TFV resulted in a variation of reflectivity angle of ΔRIU = 15 μRIU with respect to the non-specific interaction and ΔRIU = 22 μRIU with respect to the blank buffer.

### Sensing surface optimization

**Figure 2:**
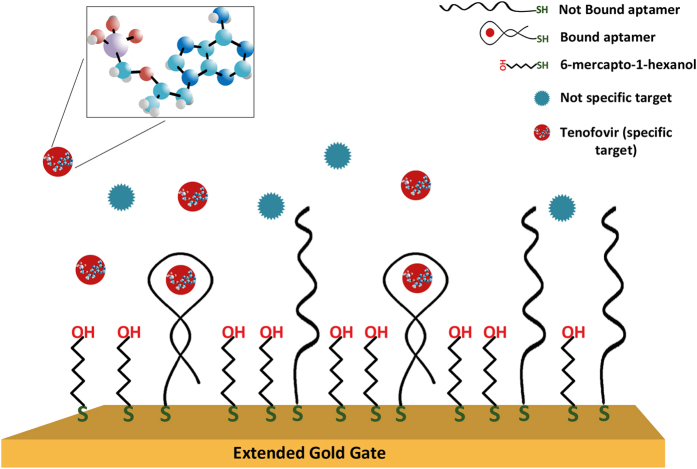
Binary SAM of TFV-aptamer and MCH as sensing surface of biosensor. In the inset: chemical structure of TFV with red color for Oxygen, purple for phosphorus, light blue for carbon, white for hydrogen and dark blue for nitrogen.

**Figure 3:**
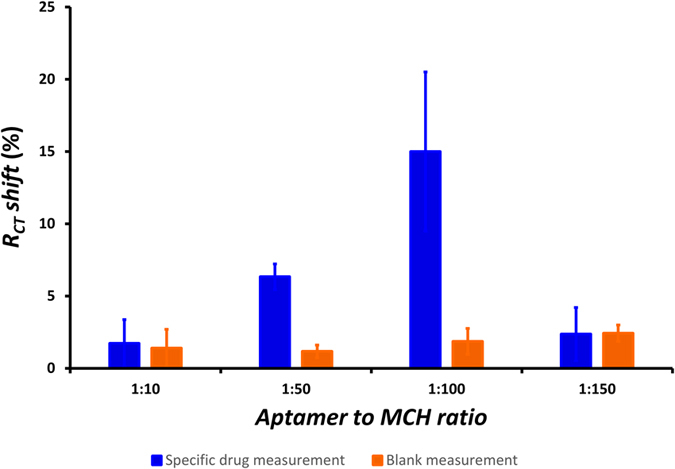
Sensing surface optimization. Blue bars indicate EIS responses of AptaFET to 500 nM of TFV exploiting various aptamer to MCH ratios. Highest RCT shift for 1:100 ratio. Error bars in this and following Figures represent the standard error of triplicate measurements carried out on three electrodes.

The sensing surface was optimized by using electrochemical impedance spectroscopy (EIS). A schematic diagram of the sensing interface, containing the chemical structure of TFV is presented in Fig. 2. Several sensing surfaces were prepared with 1:10, 1:50, 1:100 and 1:150 ratios of thiolated aptamer to total thiol molecules (aptamer + MCH), and tested by EIS in the presence of 500 nM of TFV. Upon binding of the aptamer to the drug, the aptamer undergoes a conformational change, increasing the charge density closer to the electrode and hence increasing the electrostatic potential barrier to the negativelly charged redox marker – i.e. an increase in R_ct_ is observed. The average of R_ct_ shifts for each aptamer:MCH ratio is presented in Fig. 3. It is observed that the optimized ratio in terms of specific response is 1:100, showing 15.0 ± 5.5% of R_ct_ shift with respect to the baseline (blank measurement). This value is ~2.4 times higher than 6.3 ± 0.9% of shift corresponding to the 1:50 ratio. Although in this experiment a large deviation was observed in the measured response, the trend of the recorded responses was the same for all samples and following the averaged signal. The optimized ratio of 1:100 achieved in this work is consistent with results reported by Formisano *et al*.^23^ for a prostate specific antigen aptamer (PSA-aptamer):MCH binary layer. This PSA-aptamer had a similar length (32 nucleotides) and a two-dimensional structure as the TFV-aptamer used in our work. They have also reported a surface density of aptamers equal to 1 × 10^12^ molecules cm^−2^ for a similar sensing surface formed on a gold electrode of the same size and shape as in this work.

### Dose-response behavior

**Figure 4:**
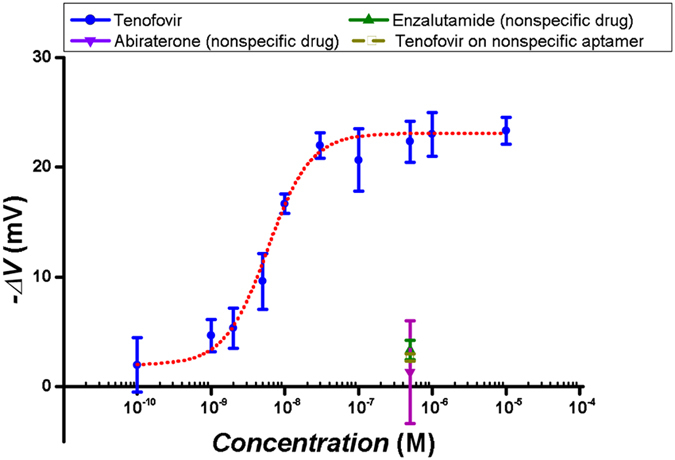
Dose-response curve of AptaFET biosensor. Specific response (blue points) fitted to hill function with linear range between 1 nM and 100 nM, and EC_50_ of 5.8 ± 0.5 nM. Responses related to non-specific drugs and non-specific aptamers as negative controls in purple green and yellow colour.

In order to study the sensing performance of the system, the AptaFET biosensor was used to detect various concentrations of TFV. The incubation procedure and other experimental details are provided in the Apparatus and setup section. In addition, the same experiment was carried out using 500 nM of both Abi and enzalutamide (Enza) solutions as negative controls on separate electrodes (without having preceeding reaction with TFV) to examine the selectivity of the AptaFET for TFV. In order to study the interaction of TFV with a non-specific sensing surface, electrodes were functionalized with a binary layer of PSA-aptamer and MCH (1:100 ratio) and tested with a 500 nM TFV solution. The dose-response curve of the AptaFET (averaged voltage shift responses versus logarithm of concentrations) is shown in Fig. 4. Controls are also presented in the figure for comparison. The curve followed a sigmoidal behavior with a linear range between 1 nM and 100 nM, and showed saturation after 100 nM. A limit of detection of 1.2 nM was calculated for the biosensor following the method described by Armbruster *et al*.^24^. Abi, Enza and PSA-aptamer experiments exhibited 1.3 ± 4.7 mV, 3.3 ± 0.9 mV and 2.7 ± 0.3 mV of I_d_-V_gs_ shift, respectively. These are negligible when compared to the 22.3 ± 1.8 mV shift obtained for TFV at the same concentration (500 nM).

### Detection of TFV in human plasma

**Figure 5:**
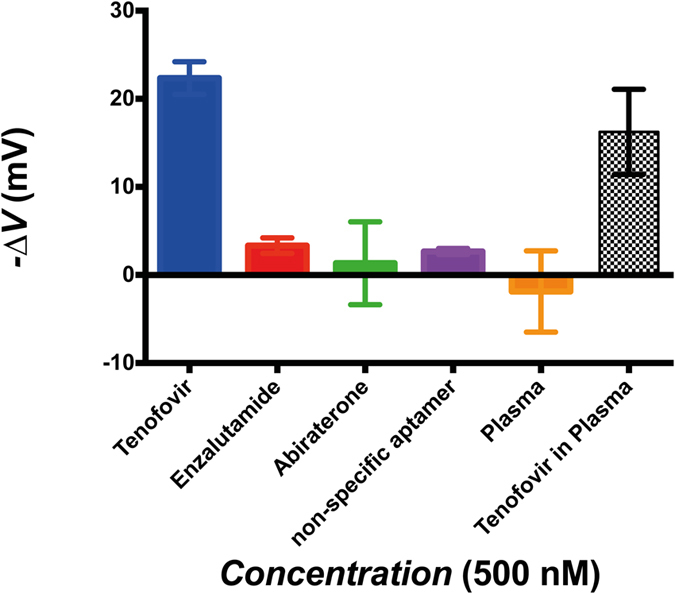
AptaFET response comparison. Response of biosensor to TFV in PBS (blue), in human plasma (black patterned). Non-specific response to negative controls: Enza (red), Abi (green) and blank blood plasma (orange). Non-specific response of TFV and PSA-aptamer interaction (purple bar). All target concentrations are 500 nM.

The performance of the TFV AptaFET was also assessed using human blood plasma. First, the interference of non-specific coexisting proteins of human serum on the biosensor was measured. To perform the experiment biofunctionalized electrodes were incubated with blank plasma (after stabilizing and taking the baseline in buffer) for half an hour and tested in PBS to obtain the response of plasma proteins and biolayer interactions. This interaction caused either small positive or small negative shifts as output, depending on the electrode, with an average response of −1.9 ± 4.6 mV. Then the electrodes were incubated in human plasma spiked with 500 nM of TFV for 30 minutes, and measurements performed in plasma. TFV binding reaction in human plasma produced a response of 16.3 ± 4.8 mV with respect to the baseline in plasma – see Fig. 5.

### Application of the biosensor in real-time monitoring

**Figure 6:**
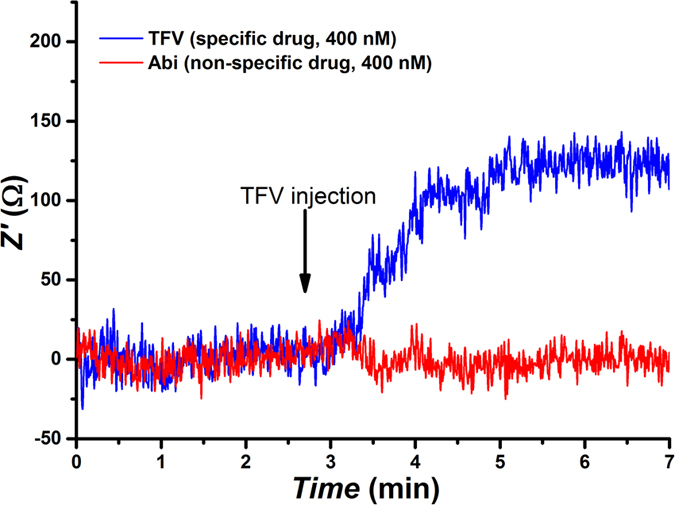
Real-time monitoring of the real part of the impedance at 10 Hz for an electrode modified with TFV aptamer upon injection of TFV as the specific target (blue) and Abi as non-specific target (red). Baselines were subtracted for better comparison.

Real-time monitoring of the drug was also assessed by measuring the specific binding reaction at the sensing surface in flow conditions. Real-time impedance measurements were used to investigate the TFV−aptamer interaction. Cleaned gold electrodes were functionalized with the optimized ratio of aptamer:MCH. The real part (Z’) of the impedance at 10 Hz was then monitored in a flow of 10 mM ferro/ferricyanide solution. Once a stable signal was observed, TFV (in 10 mM ferro/ferricyanide) was injected to the flow to a final concentration of 400 nM. Z’ started to increase after some seconds, and it stabilized at 123 ± 16 Ω after 2 minutes. The same experiment was performed using Abi as a non-specific target, which yielded null resistance increment in flow conditions. The results are shown in Fig. 6 after baseline correction.

## Discussion

TFV is classified as a nucleotide analogue reverse transcriptase inhibitor (NRTI). Reverse transcriptase is a crucial viral enzyme in retroviruses such as HIV and hepatitis-B virus infections^25^. Based on the pharmacokinetics data reported by Anton *et al*.^26^, after oral administration of TFV the circulatory plasma concentration is in the range of 20 nM to 860 nM.

In order to achieve good sensitivity and specificity of our TFV aptasensor, an optimization of the sensor surface is fundamental. Changing the ratio of aptamer to MCH in the immobilization solution gives the possibility to modify the surface coverage of the aptamer and alter the interaction between the molecules on the surface. A balance is required between the amount of aptamers available on the surface and avoiding steric hindrance effects due to the conformational changes of the aptamer required for the binding. This helps increase the sensitivity and selectivity of the biosensor towards TFV, providing low enough LOD and minimum non-specific interactions. The EIS response presented in Fig. 3 shows a signal loss with either high and low aptamer to MCH ratio. This suggests that too high a quantity of aptamers immobilised on the surface will prevent the binding and folding due to steric hindrance as well as strong electrostatic repulsion between the aptamers. This will reduce the response amplitude of the system in terms of R_ct_ variation or lower I_d_-V_gs_ characteristic shift for field-effect measurements. On the other hand, when there are too few aptamers on the surface, there is low TFV binding and reduction of the response. The latter also increases the possibility of non-specific response of the system due to possible interactions of proteins with MCH.

Real-time monitoring impedance experiments (Fig. 6) show the quality of TFV binding to the specific aptamer, and minimum non-specific interferences in flow conditions, demonstrating the capability of the biosensor to be exploited in real-time drug monitoring.

As presented in Fig. 5, high selectivity is achieved for the AptaFETs, both in PBS buffer and in blood plasma. Negligible non-specific binding responses are observed for controls such as Enza and Abi, and also for the interaction of target with non-specific sensing surfaces (anti-PSA aptamer). The data shows a comparable AptaFET response for TFV detection in blood plasma to the one obtained in buffer. Exposure of the sensing surface to blank human plasma shows high resistance to fouling by plasma proteins, since the response of the biosensor to non-specific plasma proteins was negligible compared to the specific response to the drug (orange bar in Fig. 5). In addition, there is the possibility of exploiting the same detection system for a wide range of targets just by exchanging the TFV-aptamer with an aptamer specific to the new target, thanks to the low non-specific binding reactions between the biomolecules in the sample and the sensing surface. This has been reported before for other types of aptamer-based biosensors^4^.

The small positive and negative shifts of the FET output as response to plasma interaction was expected, since blood plasma contains various ranges of proteins with different charges that can bind to the surface and modulate the channel in different and contradictory ways. Moreover, Abi has also presented opposite signal shifts, which is due to the nature of its chemical structure; different interactions with the SAM layer causes different channel modulation and, thus, opposite signal shift of the output.

The calculated LOD of the AptaFET is 1.2 nM, which is lower than what has been reported with usual electrochemical sensors, for example, on cytochromes P450^13^. This shows the extremely good performance of this novel AptaFET for applications such as a TFV monitoring system. The LOD of the AptaFET falls well within the therapeutic range of TFV. In addition, the dose-response curve (Fig. 4) exhibits a highly sensitive linear range between 1 nM and 100 nM. The linear range of the AptaFET is lower than the therapeutic range of the target, and the system saturates before the maximum of the range (860 nM). This difference is an advantage for drug detection in human fluids, as it gives the possibility of sample dilution that will decrease the non-specific interactions and interferences related to the various metabolites that coexist in human plasma. To compare non-specific and specific responses, a high concentration of a non-specific drug was used to show that even at such high concentrations the response of the biosensor is negligible. The aptamer used in this work has been characterized by Kammer *et al*.^27^ using backscattering interferometry with a K_d_ value of 9 ± 1.4 nM. They presented the specificity of the aptamer to its target by using penicillin as a negative control. The response of the AptaFET presented in this work is consistent with the data published by Kammer *et al*. The EC_50_ = 5.8 ± 0.5 nM, which is calculated based on the dose-response curve for the biosensing surface, alongside the K_d_ value of 9 ± 1.4 nM^27^ demonstrate a high affinity of the aptamer towards TFV and, thus, strong drug-aptamer binding. The possibility of loosing bonded drugs without an extensive effort is very low. Nevertheless, we expect a small amount of drug dissociation and association on the surface that reaches equilibrium and does not produce significant interferences in biosensor response. A list of recent publications (after 2013) on small molecule monitoring is summarized in Tables 1 and 2. The performance of the AptaFET in terms of LOD and linear range is better or comparable to other biosensors that are suitable for POC analytical applications (Table 1), or even the ones utilizing very complex and time consuming sample preparation or complex analytical processes (Table 2). These techniques are less suitable or not suitable at all for POC drug monitoring. For instance, fluorescent-based analytical methods need laboratory-labeling processes, which are complex and can interfere with the functionality of the biomolecule. Some label-free optical and mass spectrometry methods require professionals for operation or their application is limited to a specific class of biomolecules (not all the molecules are ionizable). Although low LODs have been obtained for some small molecules using different techniques, the LOD of this work is the lowest in the literature for TFV detection and fully suitable for clinical applications. Comparing the data presented in this work with the literature shows the main advantage of our novel AptaFET with respect to many of the reported small molecule analytical methods suitable for POC applications. This work compared quite well with other works in literature^4^,^21^,^28^,^29^ in terms of sensitivity and selectivity (see Table 1). It works very well in human plasma with an extremely good detection and capability to provide real-time monitoring.

Dealing with the issue of selectivity of the presented sensor, we have also observed with SPR some non-specific binding when fluxing Abi on the sensing surface (Fig. 1). This non-specific interaction was expected, considering the chemical structure of Abi and the molecules on the surface. The Abi structure harbours three main features, including the aromatic nitrogen-containing heterocycle (pyridine moiety), the hydrophobic steroidal core, and the hydroxyl group that together increase the non-specific binding of Abi through hydrophobic forces or hydrogen binding to electronegative species that exist in the aptamer structure and on the MCH^30^,^31^,^32^. However, during the dissociation cycle, when the surface was washed with blank buffer, the majority of Abi molecules were washed away, while the TFV molecules maintained their uptake. This caused the Abi signal to be 15 μRIU lower than for TFV at the dissociation cycle.

In conclusion, this work proposed and characterized a POC drug monitoring method suitable for detection in human plasma and real-time monitoring. The LOD and linear range of the biosensor were fit to the physiological ranges of the target. The sensitivity and selectivity of the biosensor towards the specific target was successfully tested. In addition, this method can be used for monitoring of other biomolecules by exchanging the TFV-aptamer with another aptamer that is specifically produced for the new target. These prove the capability of the proposed detection method to be utilized for continuous monitoring of a wide range of small molecules in human plasma.

## Materials and Methods

### Materials

Thiolated TFV-aptamers (5′-Aptamer-C6 Thiol-3′) were commercially provided by BasePair Biotechnologies (Pearland, Houston, USA). Thiolated PSA-aptamer (HS-(CH_2_)_6_–5′ TTT TTA ATT AAA GCT CGC CAT CAA ATA GCT TT 3′), human plasma, MCH, potassium phosphate monobasic solution (KH_2_PO_4_), potassium phosphate dibasic solution (K_2_HPO_4_), potassium sulphate (K_2_SO_4_), potassium hexacyanoferrate (III), potassium hexacyanoferrate (II) trihydrate, magnesium chloride (MgCl_2_), DMSO and PBS (10 mM; pH 7.4) were all purchased from Sigma-Aldrich (UK). Aptamers were unfolded by heating them at 90 °C for 5 minutes and letting them cool down to room temperature. TFV, Abi and Enza powders were purchased from Medchemtronica (Stockholm, Sweden) and were dissolved in dimethyl sulfoxide (DMSO) to the concentration of 5 mM. PB (0.1 M pH 7.0) was prepared by adding 17 mM of KH_2_PO_4_, 33 mM of K_2_HPO_4_ and 500 nM of K_2_SO_4_ to distilled water. All other reagents were of analytical grade. All aqueous solutions were prepared using 18.2 MΩ cm ultrapure water with a Pyrogard filter (Millipore, Feltham, UK).

### Biosensor development

The procedure to find out the best sensing surface in terms of the sensitivity, selectivity and level of complexity is explained briefly in the Supplementary information. The focus of the optimization was on improving the performance of the tenofovir-aptamer interaction to enhance the specificity of the response. In addition, stability and reliability of the biosensor was tested using different chemistries for building the biorecognition surface. Several different sensing surfaces were tested until finally a binary self-assembled layer of TFV-aptamer and MCH was chosen to be fabricated on the interface as sensing surfaces (Figures S4 to S7). The aptamer was developed for specific interaction with TFV, while MCH was used as a surface blocking-agent and spacer between aptamers (Fig. 2). Gold disk working electrodes (radius 1.0 mm, CH Instruments, Austin, TX, USA) were cleaned based on the procedure published by Keighley *et al*. to provide a properly flat and contaminant free surface for biomolecule immobilization^33^. The gold electrodes were then incubated in a solution of tenofovir-aptamer and MCH in PB buffer for 16 hours to form the biosensing surface. This step was followed by 50 minutes of 1 mM MCH backfilling to complete the surface blockage and lifting any aptamers lying down on the surface^23^. These bioelectrodes were used in EIS experiments for sensing surface optimization. The immobilization solution consisted of 1 μM of aptamer and 10, 50, 100 and 150 μM of MCH in this experiment to obtain different aptamer surface coverage (1:10, 1:50, 1:100 and 1:150 ratios of aptamer:MCH, respectively).

Our innovative AptaFET biosensor consists of a home-designed n-MOSFET structure combined with an extended gate (more details in Supplementary material, including the schematic in Figure S3). Detailed information on the specifications of the n-MOS chip has been reported elsewhere^16^. Arrays of gold electrodes (180 nm Au thickness on 20 nm Cr) were deposited on a glass substrate using thermal evaporation. Electrodes were cleaned by acetone, ethanol and ultrapure water, sequentially and repeatedly, and then they were functionalized with the aptamer as described earlier. The extended gate was fabricated by connecting the biofunctionalized Au electrodes, fixed in a reaction cell, to the gate of an n-MOSFET via a metal wire.

### Apparatus and setup

Surface plasmon resonance (SPR) measurements were performed using a Reichert SPR 7000DC (USA) dual channel flow spectrometer at 25 °C. 50 nm gold coated SPR gold chips, supplied from Reichert Technologies were used for studying the reaction on SPR. Prior to their modification, the chips were cleaned using piranha solution (3:1 H_2_SO_4_:H_2_O_2_) for 20 seconds and rinsed thoroughly with MilliQ water and dried using nitrogen gas. Modification of the SPR chips with DNA aptamers was performed in the same way as described earlier. All buffers were filtered through 0.2 μm filters and degassed for 2 h by sonication prior to the experiment. To perform the experiment, 500 nM solutions of TFV and Abi were flowed over the biofunctionalized chips for 20 minutes with a flow rate of 25 μL min^−1^, and followed by 5 minutes of a dissociation step to remove all the unbound residues. For the EIS experiments, a μAUTOLAB III/FRA2 potentiostat (Metrohm, Netherlands) and a three-electrode cell setup consisting of biofunctionalized gold working electrode, Ag/AgCl reference electrode (via a salt bridge filled with 0.1 M PBS pH 7.4) and platinum counter electrode (ALS, Tokyo, Japan) were used. The measurements were carried out in PBS buffer solution containing 10 mM of ferro/ferricyanide [Fe(CN)_6_]^3−/4−^ redox couple (hexacyanoferrate II/III), applying a 10 mV a.c. voltage superimposed on a +0.2 V of d.c. potential (formal potential of the redox couple), in the frequency range of 100 kHz to 0.1 Hz (61 frequency steps). After a stable signal was observed with blank solutions, the biofunctionalized electrodes were incubated in 500 nM solution of TFV for 30 minutes and washed in buffer before the EIS spectra was measured. For real time impedance monitoring, a three-electrode flow cell was connected to a CompactStat potentiostat (Ivium Technologies, The Netherlands). The impedance was monitored every 0.2 s at a single frequency of 10 Hz with a 10 mV amplitude superimposed on a 0.2 V d.c. bias potential. Solutions of TFV and Abi were injected once a stable baseline was reached to obtain the total concentration of 400 nM in the flow. To operate the field-effect measurements using the AptaFET, a V_ds_ of 50 mV was applied across the drain to source and V_gs_ was swept from 0 to 4 V. These settings limited the I_d_ to less than 75 μA to avoid any false measurement due to device heating effects. Biofunctionalized electrodes were stabilized in PBS buffer, and then incubated for 30 minutes in 100 pM, 1 nM, 2 nM, 5 nM, 10 nM, 30 nM, 100 nM, 500 nM, 1 μM and 10 μM solutions of TFV in PBS-MgCl_2_. They were then washed and measured in PBS to obtain the dose-response curve. The AptaFET was further measured with 500 nM of Abi and Enza as negative controls using the same procedure. Finally, a PSA-aptamer with the length (32 nucleotides) and two-dimensional structure similar to the TFV-aptamer was used in order to investigate the interaction of TFV with a non-specific sensing surface of the same length and structure. For the experiments on plasma samples, drugs were solved in blank human plasma to the final required drug concentrations.

### Statistical analysis

All the indicated errors in this work (including the error bars in figures) are the standard deviation of triplicate measurements performed on three separate electrodes that are produced and experimented under the same conditions. Nonlinear regression was used to fit a modified version of the Hill function (equation 1) as an empirical model to the dose response data presented in Fig. 4. This model was prefered to the normal Hill function, because it correlates the recorded effect (response of biosensor) to the binding reaction (analyte concentration), while the conventional Hill function describes the relation between the binding reaction and the concentration of the occupied binding sites^34^,^35^. The nonlinear regression analysis was performed using the fitting tools of Origin 8.5 (OriginLab, USA).


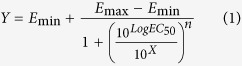


To obtain the LOD of the biosensor, first LOD_ΔV_ was calculated using equation 2 and 3,^24^, and then it was converted to concentration using the fitting equation (equation 1).


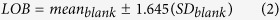



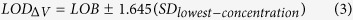


where mean_blank_ = 1.8 mV and SD_blank_ = 1.2 mV are the average and standard deviation of 3 blank measurements, respectively. SD_lowest-concentration_ = 4.3 mV is the standard deviation of the 3 responses related to the lowest concentration of TFV experimented (100 pM in this work). LOB = 3.8 mV or limit of blank, is the highest blank measurement response obtained by testing 3 blank samples. LOD_ΔV_ = 10.8 mV is the lowest response caused by TFV that is distinguishable from LOB, and can be considered as a reliable detection of the drug. EC_50_ = 5.8 ± 0.5 nM is the calculated concentration that produces half the maximum binding response. The calculations result in a LOD of 1.2 nM for the AptaFET, an extremely good performance for an electrochemical sensor. The average reproducibility measured for the dose-response curve beyond the limit of detection is equal to 36.5%.

Although the dissociation constant (K_d_) can be considered to be roughly equal to EC_50_, it is not possible to directly calculate its exact value using the fitting model of this work^35^.

## Additional Information

**How to cite this article**: Aliakbarinodehi, N. *et al*. Aptamer-based Field-Effect Biosensor for Tenofovir Detection. *Sci. Rep.* **7**, 44409; doi: 10.1038/srep44409 (2017).

**Publisher's note:** Springer Nature remains neutral with regard to jurisdictional claims in published maps and institutional affiliations.

## Supplementary Material

Supplementary Information

## Figures and Tables

**Table 1:** Example of recent published works on small molecule detection exploiting POC-compliant detection methods.

Target	Analytical method^†^	LOD	Linear range	References
Acetaminophen	Amperometry and DPV	2.1 nM	5 nM to 800 μM	36
Adenosine	EIS	20 fM	0.05 pM to 17 pM	37
Bisphenol A	FET	56 pM	1 to 10^4^ fM	38
Chloramphenicol	SWV	5 nM	40 to 1000 nM	39
Cocaine	DPV	0.13 nM	0.1 to 10 nM	40
17β-estradiol	FET	50 nM	50 nM to 1.6 μM	28
Etoposide	DPV	5.4 nM	20 nM to 2 μM	41
Etoposide	SWV	1.29 μM	10 to 60 μM	42
Glucose	SWV	4 mM	4 to 20 mM	43
Glucose	FET	2 mM	2 to 8 mM	44
Kanamycin	SWV	14 pM	10 nM to 2 μM	45
Naproxen	CV	16 μM	Up to 300 μM	12
Oxytetracycline	SWV	0.22 nM	1.1 pM to 110 nM	46
Rifampicin	Amperometry	50 nM	2 to 14 μM	47
Streptomycin	SWV	10 nM	50 to 1000 nM	39
**Tenofovir**	**FET**	**1.2 nM**	**1 nM to 100 nM**	**This work**
Tenofovir	SWCAdSV	1.3 μM	1.7 to 17.4 μM	48
Theophylline	CV	50 nM	Up to 120 μM	49

^†^CV = Cyclic Voltammetry; SWV = square wave voltammetry; FET = field effect transistor; EIS = electrochemical impedance spectroscopy; DPV = differential pulse voltammetry; EMAT = electromagnetic acoustic transducer; LSV = linear sweep voltammetry; SWCAdSV = square-wave cathodic adsorptive stripping voltammetry.

**Table 2:** Example of recent published works on small molecule detection exploiting methods that are not compliant to POC drug monitoring.

Target	Analytical method^†^	LOD	Linear range	References
Acetaminophen	UV-Vis spectroscopy	1.8 μM	6 to 38 μM	50
Adenosine	Fluorescent spectroscopy	3 μM	1 to 25 μM	51
Bisphenol A	Fluorescent spectroscopy	8 nM	44 to 350 nM	52
Diclophenac	Reflectometric interference spectroscopy	0.4 nM	0.4 nM to 3.4 nM	53
Etoposide	Fluorescent spectroscopy	850 nM	850 nM to 34 μM	54
Glucose	UV-Vis spectroscopy	10 nM	—	55
Kanamycin	Fluorescent spectroscopy	0.44 nM	0.5 to 20 nM	56
Naproxen	UV and Fluorescent spectroscopy	0.9 nM	—	57
Oxytetracycline	Cantilever deflection	0.2 nM	10 to 100 nM	58
Tenofovir	Mass spectroscopy	0.87 nM	3.5 to 3500 nM	59
**Tenofovir**^**‡**^	**FET**	**1.2 nM**	**1 nM to 100 nM**	**This work**

^†^UV = ultraviolet; Vis = visible; BSI = backscattering interferometry.

^‡^This work is inserted here for comparison.
