# Supplementary material for: Aptamer-based Field-Effect Biosensor for Tenofovir Detection
Source: Sci Rep. 2017 Mar 15;7:44409. doi: 10.1038/srep44409 (PMC5353720; doi:10.1038/srep44409)
Supplement: Supplementary Information [file srep44409-s1.pdf]

SUPPLEMENTARY MATERIAL

Aptamer-based Field-Effect Biosensor for Tenofovir Detection

N. Aliakbarinodehi[1]*, P. Jolly[2], N. Bhalla[2†], A. Miodek[2‡], G. De Micheli[1], P. Estrela[2], S. Carrara[1]

[1] School of Engineering, École Polytechnique Fédérale de Lausanne (EPFL), STI-IEL-LSI2, Building INF, 3rd floor, 1015 Lausanne, Switzerland.
[2] Department of Electronic and Electrical Engineering, University of Bath, Claverton Down, BA2 7AY Bath, United Kingdom.
[†] New affiliation: Micro/Bio/Nanofluidics Unit, Okinawa Institute of Science and Technology, 1919-1 Tancha, Onna-son, Kunigami-gun Okinawa, 904-0495, Japan
[‡] New affiliation: Alternative Energies and Atomic Energy Commission (CEA), Institute of Biomedical Imaging (I^2BM), Molecular Imaging Research Center (MIRCen), 18 Route du Panorama, 92265 Fontenay-aux-Roses, France
[*] Corresponding author. Email: nima.aliakbarinodehi@epfl.ch

Investigating the sensing surface and TFV interaction

Square wave voltammetry (SWV) and intercalator, daunomycin, were exploited in order to support the data presented in this work by showing that the responses of EIS and FET experiments were caused by binding of drug and aptamer conformational changes. To this end, the SWV response of the sensing surface was recorded after daunomycin intercalation following three different orders:

A) Daunomycin intercalation (no drug bound to aptamer)
B) Sensing surface incubation in TFV solution, then daunomycin intercalation
C) Daunomycin intercalation, then sensing surface incubation in TFV

The recorded SWV responses (Figure S1) were analyzed in Nova software and the peak amplitudes are illustrated in Figure S2 for comparison.

Intercalators bind to DNA due to two phenomena: first, intercalation to hydrophobic state of a double stranded DNA (dsDNA); second, electrostatic interactions with phosphate groups of the DNA backbone[1]. Therefore, intercalation happens to both single stranded DNA (ssDNA; aptamers before binding and conformational change) and dsDNA (part of aptamer after conformational change) to some extent. It has been reported in the literature that binding is more effective in dsDNA than in ssDNA [1,2].

The conformational change of the TFV aptamers after the incubation with drug can be inferred from the data presented in Figure S2, suggesting the successful binding phenomena. The average of the oxidation peaks was higher in the case where daunomycin was intercalated with the aptamers that have already been incubated in specific drug solution (B) than in the case where they were not (A). Further proof is the SWV response showing that the oxidation peaks were increased when the same electrodes of case A were incubated in drug solution (case C). This shows that the binding reaction had caused a conformational change in aptamer structure that brought the daunomycin molecules, which are already intercalated to the structure, closer to the electrode surface.

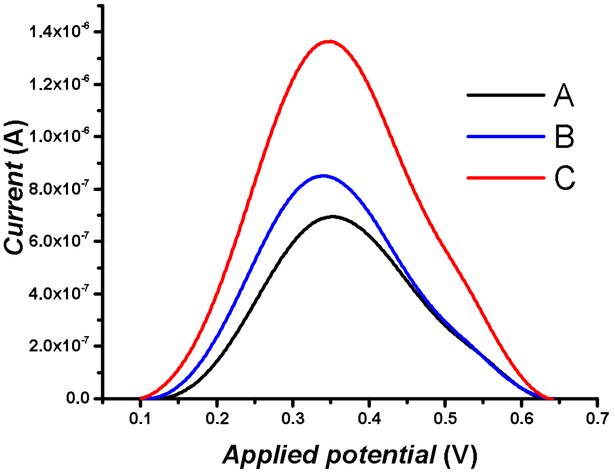

Fig S1. Oxidation peak of daunomycin for: sensing surface without any drug incubation (A, black); with drug incubation (B, blue); with drug incubation after intercalation (C, red)

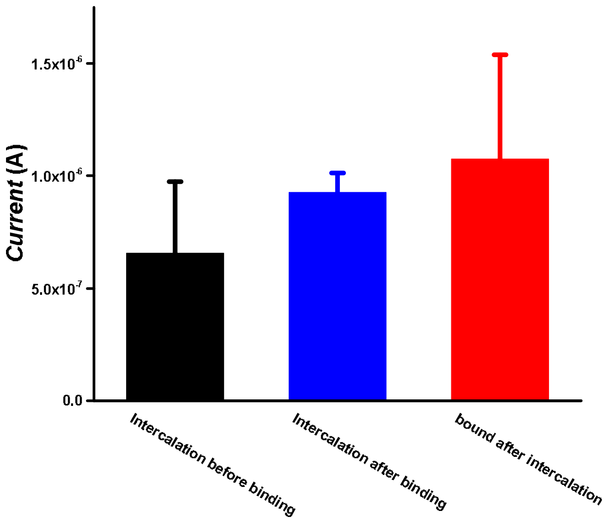

Fig S2. Average amplitude of SWV responses of daunomycin for sensing surface without any drug incubation (Black); incubated in TFV solution (blue); incubated in TFV solution after intercalation with daunomycin

Field Effect Transistor as signal transducer

FETs have attracted much attention as they offer rapid, label-free and cost effect detection of the analyte. In digital electronics MOSFET acts as a switch where two electrodes (source and drain) are used to connect a semiconductor material (channel). Current flowing through the channel between the source and drain is electrostatically controlled by a third electrode (gate), which is capacitively coupled through a dielectric layer covering the channel region[3]. In the case of an extended-gate MOSFET biosensor the physical gate is extended by connecting it to an external electrode functionalized with specific receptors for selective capturing of the desired biomolecules. In this work a home designed n-MOSFET device has been used as the signal transducer that in combination with the gold extended gates, biofunctionalized with TFV-aptamers, formed the AptaFET biosensor specific for TFV detection. A schematic of the biosensor is illustrated in Figure S3.

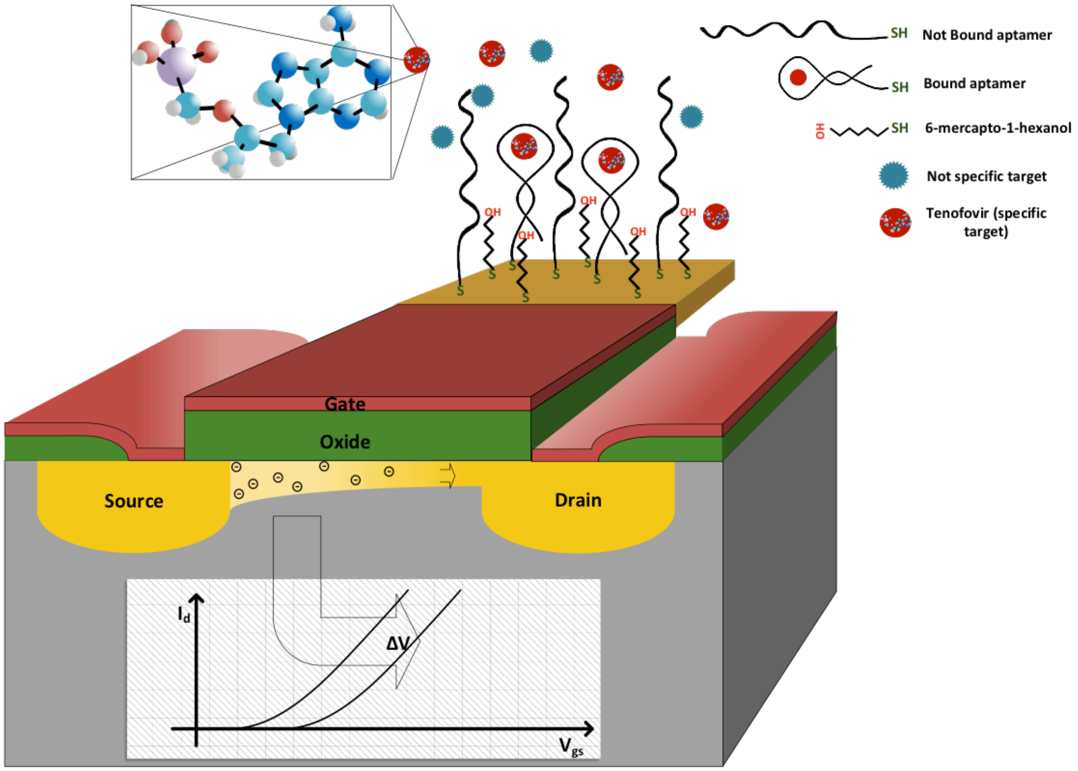

Fig S3. Schematic of AptaFET. Binding reaction is transformed to electric signal through FET and observed as I_d-V_{gs} shift. TFV is indicated by red circle.

The detail of the structure and MOSFET production is mentioned elsewhere[4]. The charged biomolecules when captured by probe molecules produce a gating (electrostatic) effect, which is transduced into a readable signal in the form of change in electrical characteristics of the MOSFET such as drain-to-source current or channel conductance[3,5]. Some applications where MOSFETs are employed for biosensing include cancer therapy[6], drug screening[7,8], POC disease detection[9] and diabetes management[10].

Biosensing surface optimization

The biosensing surface used in this work was obtained after a range of optimization experiments. The focus of the optimization was to improve the performance of the tenofovir-aptamer interaction to enhance the specificity of the response. In addition, stability and reliability of the biosensor was tested using different chemistries for the biosensing surface. To do so, different sensing surfaces were formed and tested by EIS. Specific target of the aptamer (tenofovir) was used to examine the specific response and abiraterone was exploited to examine the nonspecific response. Final results of these experiment presented that the binary SAM of aptamer:MCH provides the best performance.

The following sensing surfaces were examined in this optimization step:

1. SAM layer: ternary SAM of aptamer, MCH, hexanedithiol (HDT)

   Sensing surface includes a SAM of thiolated aptamer: 1,6-hexanedithiol (ratio: 1-3000) and backfilling with MCH. EIS responses to blank solution (Figure S4, right) presented instabilities.

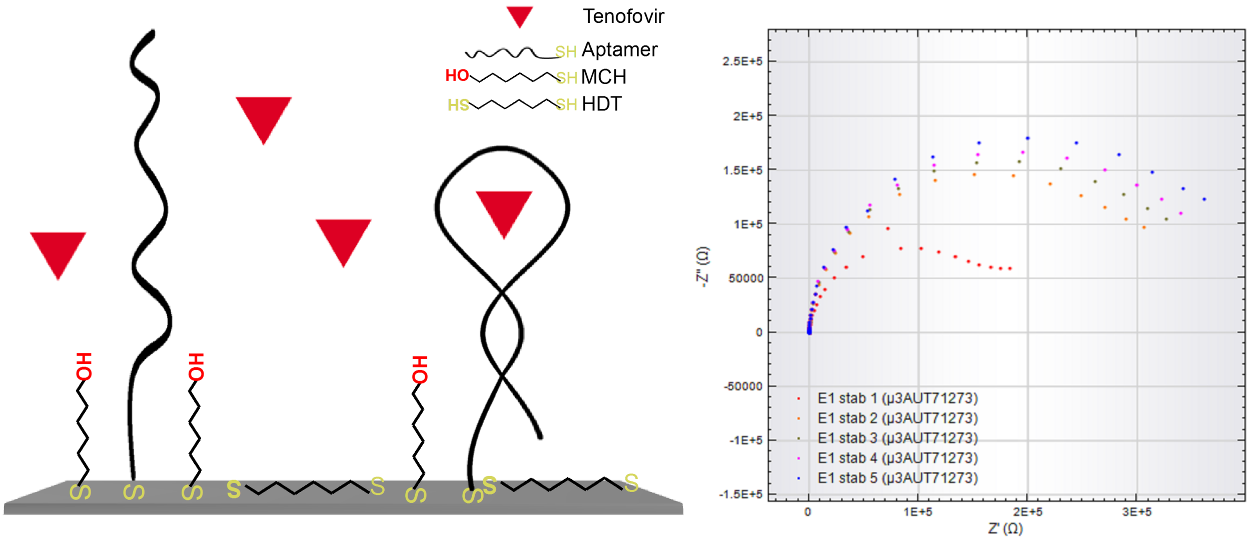

Fig S4. Schematic of sensing surface including ternary SAM of aptamer, HDT and MCH (left), an example of EIS response of the sensing surface in blank solution (right).

2. SAM layer: mercaptopropainic acid (MPA)-mercaptopropanol (MP)

   Sensing surface includes a SAM of MPA and MP (tested ratios: 1:50, 1:200), and then aptamers were immobilized over dendrimer (4th generation) through glutaraldehyde. Finally, ethanolamine was used for backfilling. EIS responses to specific target and negative control were of the same order, showing low specificity of the sensing surface (Figure S5-Right).

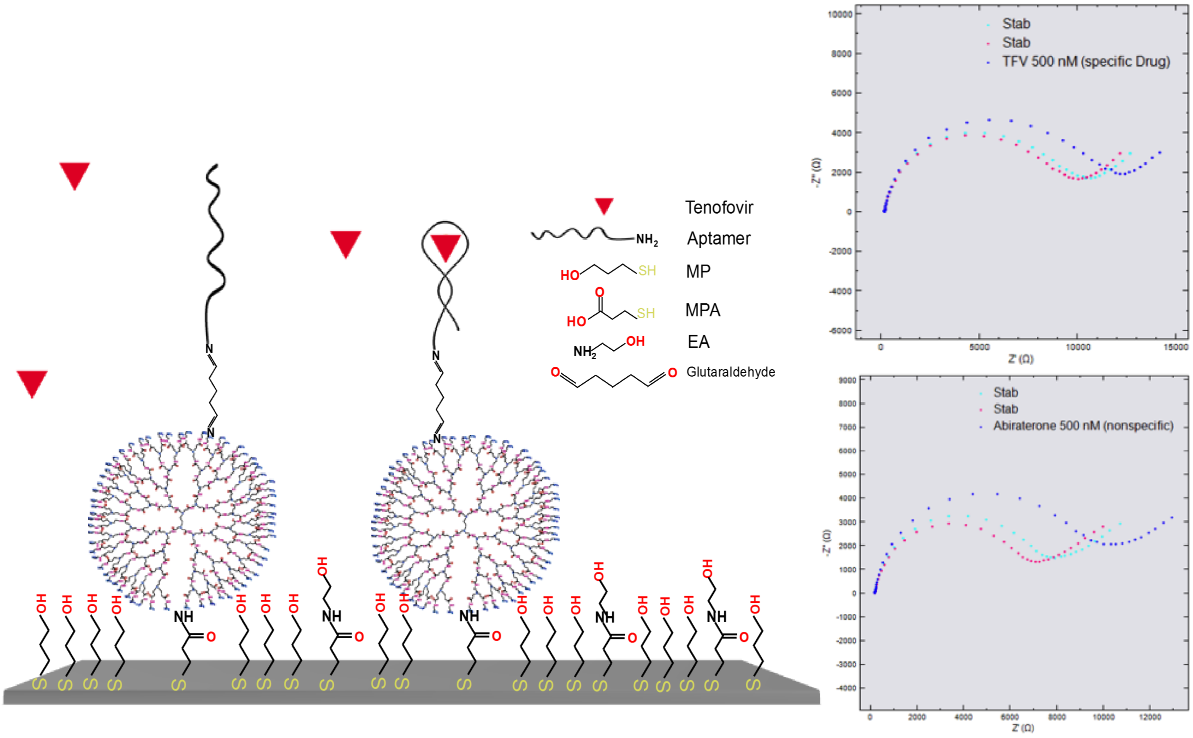

Fig S5. Left: schematic of sensing surface including aptamers over a layer of dendrimers that were immobilized over a SAM of MPA and MP. Right-top: an example of EIS response of the sensing surface to 500 nM TFV as specific target. Right-down: EIS response of the sensing surface to 500 nM abiraterone as negative control.

3. SAM layer: mercaptooctanoic acid (MOA)-MCH

Sensing surface includes a SAM of MOA and MCH (tested ratios: 1:10, 1:50, 1:100), and then aptamers were immobilized over dendrimer (4th generation) through glutaraldehyde. Finally, ethanolamine was used for backfilling.

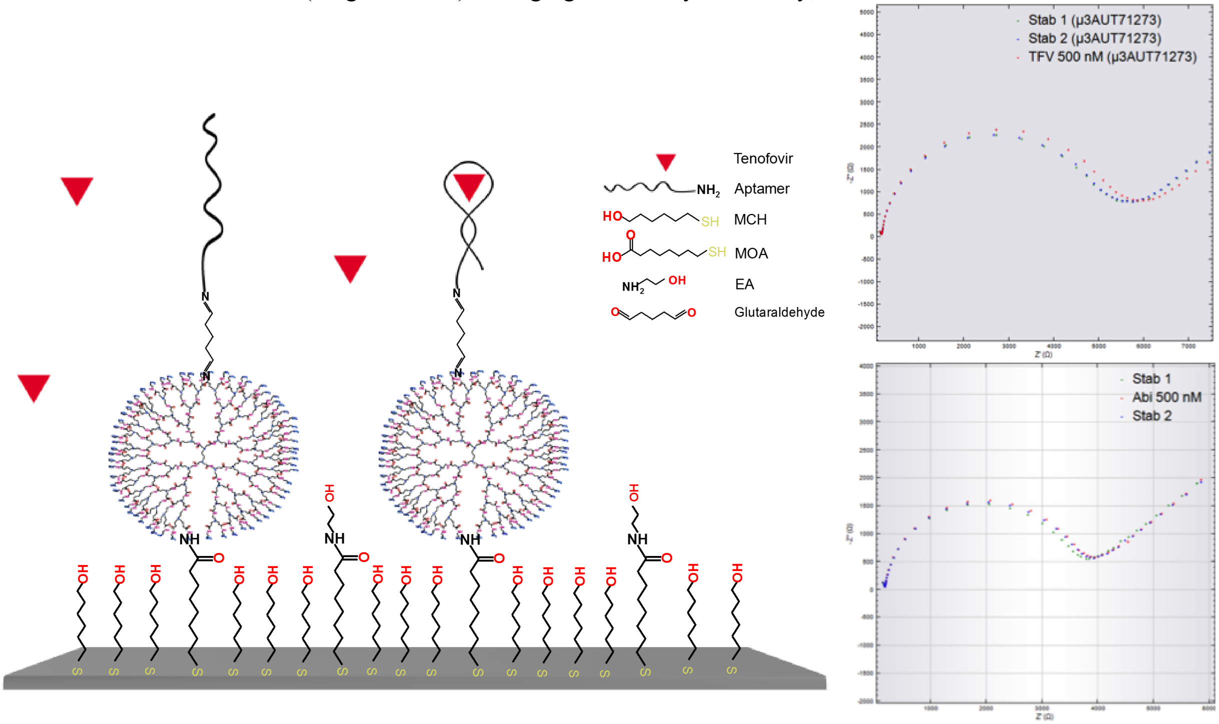

Fig S6. Left: schematic of sensing surface including aptamers over a layer of dendrimers that were immobilized over a SAM of MOA and MCH. Right-top: an example of EIS response of the sensing surface to 500 nM TFV as specific target. Right-down: EIS response of the sensing surface to 500 nM abiraterone as negative control.

EIS responses to negative control (Figure S6-Right-down) were null, and it responded to specific target approximately 10% of signal shift (Figure S6-Right-top). This proved the stability and specificity of this sensing surface. However, it was very complex and time consuming to form this surface.

4. SAM layer: binary SAM of aptamer-MCH

Sensing surface includes a binary SAM of aptamer and MCH (tested ratios: 1:10, 1:50, 1:100, 1:150). EIS responses to negative control (Figure S7-f) were null, and it responded to the specific target with ~15% of average signal shift (Figure S7-b, c, d, e). This biosensing surface exhibited higher sensitivity, higher specificity, and it saved time and effort as well.

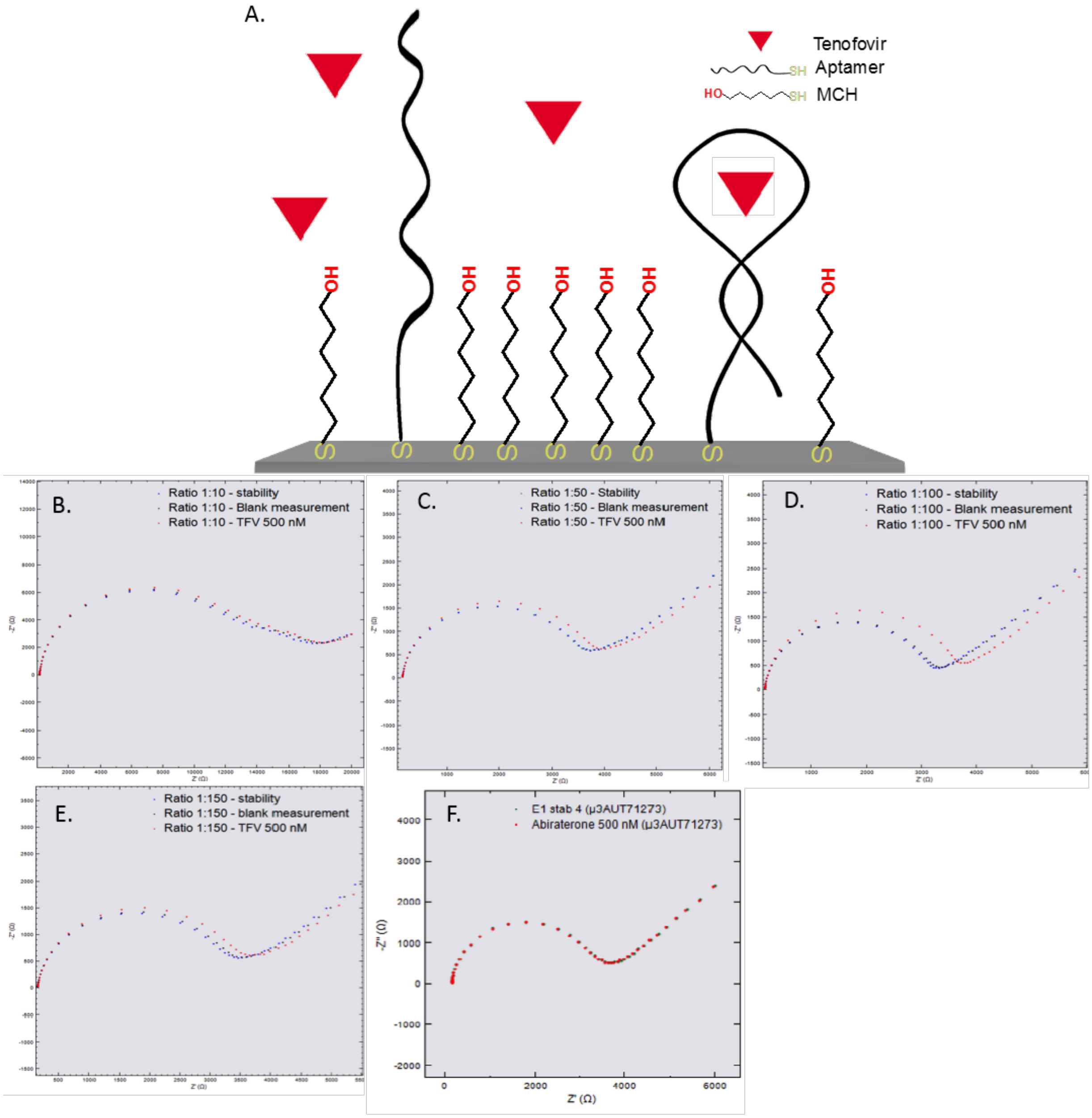

Fig S7. Schematic of sensing surface including binary SAM of aptamer and MCH (A). Response of sensing surface with ratio of 1:10, 1:50, 1:100, and 1:150 to 500 nM TFV as specific target (B, C, D, E respectively). EIS response of the sensing surface to 500 nM abiraterone as negative control (F).

REFERENCES

1. Hashimoto, K., Ito, K. & Ishimori, Y. Sequence-Specific Gene Detection with a Gold Electrode Modified with DNA Probes and an Electrochemically Active Dye. *Anal. Chem.* **66,** 3830–3833 (1994).
2. Cai, H., Cao, X., Jiang, Y., He, P. & Fang, Y. Carbon nanotube-enhanced electrochemical DNA biosensor for DNA hybridization detection. *Anal. Bioanal. Chem.* **375,** 287–293 (2003).
3. Estrela, P. *et al.* Label-free sub-picomolar protein detection with field-effect transistors. *Anal. Chem.* **82,** 3531–3536 (2010).
4. Formisano, N. *et al.* Inexpensive and fast pathogenic bacteria screening using field-effect transistors. *Biosens. Bioelectron.* **85,** 103–109 (2016).
5. Chi, L.-L., Chou, J.-C., Chung, W.-Y., Sun, T.-P. & Hsiung, S.-K. Study on extended gate field effect transistor with tin oxide sensing membrane. *Mater. Chem. Phys.* **63,** 19–23 (2000).
6. Beyer, G. P. *et al.* An Implantable MOSFET Dosimeter for the Measurement of Radiation Dose in Tissue During Cancer Therapy. *IEEE Sensors J.* **8,** 38–51 (2008).
7. Freeman, R., Gill, R. & Willner, I. Following a protein kinase activity using a field-effect transistor device. *Chem. Commun.* (2007). doi:10.1039/b707677k
8. Bhalla, N., Lorenzo, M. D., Pula, G. & Estrela, P. Protein phosphorylation detection using dual-mode field-effect devices and nanoplasmonic sensors. *Sci. Rep.* **5,** (2015).
9. Tarasov, A. *et al.* A potentiometric biosensor for rapid on-site disease diagnostics. *Biosens. Bioelectron.* **79,** 669–678 (2016).
10. Ali, S. M. U., Nur, O., Willander, M. & Danielsson, B. Glucose Detection With a Commercial MOSFET Using a ZnO Nanowires Extended Gate. *IEEE Trans. Nanotechnol.* **8,** 678–683 (2009).
